# Building trusting relationships with staff members of nursing homes during rapid ethnographic research

**DOI:** 10.3389/fsoc.2022.983728

**Published:** 2022-08-24

**Authors:** Katharina Rosteius, Bram De Boer, Sandra Staudacher, Hilde Verbeek

**Affiliations:** ^1^Department of Health Services Research, Care and Public Health Research Institute, Faculty of Health, Medicine and Life Sciences, Maastricht University, Maastricht, Netherlands; ^2^Living Lab in Ageing and Long Term Care, Maastricht, Netherlands; ^3^Nursing Science, Department of Public Health, Faculty of Medicine, University of Basel, Basel, Switzerland

**Keywords:** rapid ethnography, social sciences, nursing home research, long-term care, Living Lab, trust, Green Care Farm

## Introduction

In the past years, the number of older people has grown significantly, resulting in an increased need for high-quality care. Following societal, political, and financial changes, a culture change is taking place within long-term care, shifting from a more medical- to a more psychosocial understanding of care (Finnema et al., 2000). Subsequently, care organizations developed, which radically reinvented care to better meet the needs of residents. One of these innovative nursing homes are Green Care Farms for people living with dementia, where animals and gardens are naturally incorporated into care (de Bruin et al., 2010, 2017; Hassink et al., 2020). Next to these changes in the physical environment, they focus on a more relationship-centered care approach, as well as flat organizational structures to transport their vision.

To understand how such concepts can be implemented, as well as their impact on residents, informal caregivers, and staff, research methodologies are needed that explore care organizations from a holistic perspective. One of these approaches is ethnography, rooted in the aspiration to learn about the life of foreign communities (Malinowski, 2013). By the ongoing engagement with the field during data collection and analysis, researchers aim to understand the lived reality of the group being studied (Van Maanen, 2011; Draper, 2015). Because a researcher's presence will always influence the processes and interactions of the ones being studied, researchers spend long periods in the field, longing to become “part of the furniture” (Draper, 2015, p. 39). Developing lasting relationships with the participants, as well as reflecting on their own influence usually calls for enduring stays in the field.

Confronted with time- and financial restrictions coming with long stays in the field, researchers have developed a broad spectrum of rapid research approaches (Vindrola-Padros and Vindrola-Padros, 2018). An example is a rapid ethnography, which mainly differs from traditional ethnography by a much shorter time spent in the field, ranging from days to a few weeks (Chesluk and Holmboe, 2010; Sangaramoorthy and Kroeger, 2020). Common for studies using rapid ethnography in health care is the goal to collect data that is suitable for taking action or informing service delivery (Vindrola-Padros and Vindrola-Padros, 2018). While rapid ethnography proves to be a valuable and timesaving approach to data collection, the limited amount of time challenges the development of relationships and gaining the trust of participants. Staff members, who have no prior relationship with the researcher, might perceive the researcher as investigating their way of working [also reported by Malta-Müller et al. (2020)]. Consequently, they might be hesitant to openly share their thoughts, which can significantly affect the research results. While trust is instrumental to collect data about the inner world of participants, ethnography is, at the same time, in essence, relational (Desmond, 2014). Trust develops through openness and involvement in the research and depends on the personal interrelations created between researchers and participants (Fleisher, 1998). Therefore, trust is not only instrumental for collecting data by being sufficient, if not a necessary condition for people to open up to the researcher. It is also developed over time by co-producing knowledge and hence requires time, which rapid ethnography often lacks.

With this article, we present our solution on how to overcome the described shortcomings of rapid ethnography. Our research is embedded within an interdisciplinary partnership of care organizations and educational institutions: the Living Lab in Ageing and Long-Term Care. Relying on long-lasting relationships has paved the way for researchers entering the field in a specific location and facilitated building up individual, trusting relationships, which ultimately are the key to understanding contexts, culture, and mechanisms of change.

## Building on pre-existing relationships

The Living Lab in Ageing and Long-Term Care was founded in 1998 in Limburg, South Netherlands (Verbeek et al., 2020). Starting as a collaboration between a university and a nursing home, it has grown to a partnership of four educational institutions and nine long-term care providers. Today, the collaboration covers over 180 long-term care facilities and professional home care, where approximately 27,000 care staff take care of about 50,000 clients. Furthermore, the Living Lab also strives to collaborate with additional care providers, who are also outside the geographical scope of the province.

The relationships that developed during our research on Green Care Farms are a practical illustration of how these can lead to trust and can facilitate future collaboration. Between 2012 and 2017, the first study on Green Care Farms, which provide 24-h nursing home care for people with dementia, was conducted within the Living Lab (de Boer et al., 2017a). The study focused on the daily lives of residents on Green Care Farms in comparison with other nursing home care environments. In addition, the quality of care and experiences of caregivers were assessed. Findings indicated that Green Care Farms present a valuable alternative to traditional nursing homes. Residents were more active, came outdoors more often, had more social interactions, and appeared to have a higher quality of life (de Boer et al., 2017a,b). In addition, experiences by family caregivers were also more positive compared to other types of nursing homes (de Boer et al., 2019).

Commonly, research findings originating from the Living Lab are shared with stakeholders within and outside the network, co-creating knowledge together (Smit and Hessels, 2021). The initial positive indications found on Green Care Farms led to follow-up questions concerning the successful elements and possible implementation strategies for other long-term care settings. This in turn led to follow-up projects, involving stakeholders across the country (Buist et al., 2018). Being convinced they contribute to improving long-term care, the organizations and locations were generally eager to participate in research. In addition, participants, such as managers, care staff, and families, were asked to reflect and interpret the findings together with the research team. Such workshops led to initial contact with relevant stakeholders from care organizations, often before they were officially participating in a research project. For example, with some Green Care Farms, we have had contact since the project between 2012 and 2017, yet they are participating in a study, which started in 2021.

## Gaining trust in the field

Being able to rely on collaborations, which have been established over several years, significantly facilitated the relationship building when starting our fieldwork (Hewitt and Verbeek, 2022). We strongly believe that the individual relationships between researchers and staff members are a key element in obtaining valuable data. Staff members, in particular, are the key informants when a researcher aims to immerse in a field and understand how a care organization functions from the inside. Only when considering the researcher to be trustful, they will share their personal points of view and thoughts. Building bonds with staff members requires effort from the researcher when entering a setting and is a continuous process as the data collection proceeds. We identified several strategies, which helped us to gain the trust of staff members in the nursing homes we studied.

### Being open and naive

Before starting observations in a new department, our researchers invest a considerable amount of time to present themselves and get to know the staff members. Introducing a researcher as coming “from the university” has helped staff members to place him or her into a context, without sounding like external evaluators. Further, we noticed that being open about the research and showing them examples from field notes helped them understand that they are not personally being observed, but the general processes in the department. This is particularly important as field notes are regularly taken during or after observing situations or participating in activities. After understanding the researcher's aim, we noticed that staff members were usually keen and happy to help and to tell someone external about their work experiences.

It is commonly assumed that the development of trust depends on the degree of similarity between the researcher and the ones being studied. Walker and Hunt (2020), for example, discuss how the teaching staff readily accepted the researcher due to the researcher previous experiences as a teacher. Having the same education helped them to relate to him and they were more open. Because he remained an outsider during his observations, he describes himself as “experienced outsider.” Bucerius (2013) in turn describes how being an “inexperienced outsider” helped her to gain the trust of an all-male group of second-generation Muslim immigrants. Being different in her heritage and education and maintaining a researcher status, she was different from the group to a degree that helped them to overcome their distance; fostered by their curiosity.

Lacking an education as a nurse, one of our researchers doing fieldwork on a Green Care Farm was per definition “inexperienced” as described by Bucerius (2013). Longing to immerse in the lived reality of staff members at the farm, she strived for becoming an insider, but merely on an emotional base. Completing the above-mentioned terminology by Walker and Hunt (2020), she consequently thrived to become an “inexperienced insider”; a professional outsider but an emotional insider. Being an emotional insider, hence having a trustful, emotional connection with the staff members, allowed the researcher, for example, to be present during the informal lunch breaks, where staff members talked about their workday and how they felt. Surprisingly, being a professional outsider helped to reach the status of an emotional insider, because being a professional outsider allows asking naïve questions without sounding critical. In this sense, being inexperienced and having less similarity to the study participants enabled us to access detailed information on the daily nursing practice and the personal experiences of staff members.

In addition, being interested in their work and actively listening to their stories fostered the relationship and resulted in turning into an emotional insider. Snow et al. (1986) introduced the phrase “buddy researcher”—a researcher who behaves as a friend but maintains professional distance. This opens up the possibility to ask detailed questions about participants' line of reasoning, their actions, work life, and the atmosphere. The trust of research participants allows the researcher to access everyday life, and the privilege to participate in intimate moments like care events or during informal gatherings of staff members. At the same time, this challenges researchers, as everything the participants say is data (Edirisingha et al., 2014). However, while trust is needed to observe behavior and collect intimate details concerning the participants' lives, the researcher also has to keep a professional distance; otherwise, the objectivity might be threatened (Hammersley and Atkinson, 2019).

### Being close to different groups

Especially in nursing home environments, researchers face numerous identities, professions, power relations, and perspectives. When interacting with such different stakeholder groups, or even individuals, the researcher might need to adopt varying roles (Lecompte et al., 1999). In performing rapid ethnography, where time constraints play a major role, researchers have to make a decision on which stakeholder groups are the most promising sources of information and on which role the researcher should adopt when interacting with them. In one of the nursing homes included in our research, we discovered that certain groups of staff members seemed to have conflicts with the management, which challenged the role of our researchers.

In our experience, being close to different, even conflicting groups is a major challenge, especially during shorter stays in the field. A similar conflict was described by Russell (2005), who did fieldwork in a school. After being seen talking to teachers, she feared losing students' trust and realized that she had to build multiple relationships similarly. In our case, the management was the gatekeeper, allowing the researchers to access the nursing home. Staff members, on the other hand, are a major source of information. Being accepted by both groups is indispensable to be able to collaboratively produce knowledge and to get insider information as well as access to intimate situations. Being able to draw on the long-lasting relationships built within the Living Lab guaranteed us a leap of faith, especially from the management. Building on this, we adopted a non-threatening role and planned individual meetings with various stakeholders to hear their perspectives and experiences. Proactively planning secure and open conversations to listen to potentially conflicting groups has minimized the chances of being drawn to one's side.

## Conclusion and implications

Rapid ethnography presents a valuable alternative to regular ethnography when facing time constraints during data collection. However, spending little time in the field challenges the researcher's ability to develop personal relationships with participants, whose perspectives are key information for the research. Our experiences within the Living Lab of Ageing and Long-Term Care show how long-lasting relationships between practice and science can help to overcome these challenges. Looking back at over 25 years of collaboration, we can say that the fieldwork of our researchers is facilitated when managers, as well as staff members, are accustomed to a researcher's presence. Followed by strategies such as openness and naivety, as well as building a relationship with various groups similarly, researchers have a good chance to gain access to the personal world of participants. Therefore, we encourage researchers to experience the benefits of collaborations between research and practice, because after all, rapid, short-term ethnography might benefit from long-term relationships.

## Author contributions

This article was written by KR and BD, with assistance of SS and HV, who provided feedback and guidance. All authors contributed to the article and approved the submitted version.

## Funding

This project is funded by Meandergroep Zuid Limburg, Maastricht University and the Novartis University of Basel Excellence Scholarships for Life Sciences.

## Conflict of interest

The authors declare that the research was conducted in the absence of any commercial or financial relationships that could be construed as a potential conflict of interest.

## Publisher's note

All claims expressed in this article are solely those of the authors and do not necessarily represent those of their affiliated organizations, or those of the publisher, the editors and the reviewers. Any product that may be evaluated in this article, or claim that may be made by its manufacturer, is not guaranteed or endorsed by the publisher.

## References

[B1] Bucerius S. M. (2013). Becoming a “Trusted Outsider”: gender, ethnicity, and inequality in ethnographic research. J. Contemp. Ethnogr. 42, 690–721. 10.1177/0891241613497747

[B2] Buist Y. Verbeek H. de Boer B. de Bruin S. R. (2018). Innovating dementia care; implementing characteristics of green care farms in other long-term care settings. Int. Psychogeriatr. 30, 1057–1068. 10.1017/S1041610217002848 29335035

[B3] Chesluk B. J. Holmboe E. S. (2010). How teams work—or don't—in primary care: a field study on internal medicine practices. Health Aff. 29, 874–879. 10.1377/hlthaff.2009.1093 20439874

[B4] de Boer B. Hamers J. P. Zwakhalen S. M. Tan F. E. Beerens H. C. Verbeek H. (2017a). Green care farms as innovative nursing homes, promoting activities and social interaction for people with dementia. J. Am. Med. Dir. Assoc. 18, 40–46. 10.1016/j.jamda.2016.10.013 28012503

[B5] de Boer B. Hamers J. P. H. Zwakhalen S. M. G. Tan F. E. S. Verbeek H. (2017b). Quality of care and quality of life of people with dementia living at green care farms: a cross-sectional study. BMC Geriatr. 17, 155. 10.1186/s12877-017-0550-0 28724358 PMC5518159

[B6] de Boer B. Verbeek H. Zwakhalen S. M. G. Hamers J. P. H. (2019). Experiences of family caregivers in green care farms and other nursing home environments for people with dementia: a qualitative study. BMC Geriatr. 19, 149. 10.1186/s12877-019-1163-6 31138147 PMC6537153

[B7] de Bruin S. de Boer B. Beerens H. Buist Y. Verbeek H. (2017). Rethinking dementia care: the value of green care farming. J. Am. Med. Dir. Assoc. 18, 200–203. 10.1016/j.jamda.2016.11.018 28159468

[B8] de Bruin S. Oosting S. Van Der Zijpp A. Enders-Slegers M.-J. Schols J. (2010). The concept of green care farms for older people with dementia: an integrative framework. Dementia. 9, 79–128. 10.1177/1471301209354023

[B9] Desmond M. (2014). Relational ethnography. Theory Soc. 43, 547–579. 10.1007/s11186-014-9232-5

[B10] Draper J. (2015). Ethnography: principles, practice and potential. Nurs. Stand. 29, 36–41. 10.7748/ns.29.36.36.e8937 25942984

[B11] Edirisingha P. Aitken R. Ferguson S. (2014). “Adapting ethnography: an example of emerging relationships, building trust, and exploring complex consumer landscapes,” in Consumer Culture Theory, Arnould, E., and Thompson, C. J. (Bradford: Emerald Group Publishing Limited). 10.1108/S0885-211120140000016010

[B12] Finnema E. Dröes R.-M. Ribbe M. Van Tilburg W. (2000). A review of psychosocial models in psychogeriatrics: implications for care and research. Alzheimer Disease Assoc. Disord. 14, 68–80. 10.1097/00002093-200004000-00004 10850745

[B13] Fleisher M. S. (1998). “Ethnographers, pimps, and the company store,” in Ethnography at the Edge: Crime, Deviance, and Field Research, eds J. Ferrell and M. S. Hamm (Boston, MA: Northeastern University Press), 44–64.

[B14] Hammersley M. Atkinson P. (2019). Ethnography: Principles in Practice. Routledge.

[B15] Hassink J. Veen E. J. Pijpker R. de Bruin S. R. Van Der Meulen H. A. Plug L. B. (2020). The care farming sector in the netherlands: a reflection on its developments and promising innovations. Sustainability. 12, 3811. 10.3390/su12093811

[B16] Hewitt J. Verbeek H. (2022). From research to impact in long-term care: a lived experience trajectory by inaugural winners of the morley award. J. Am. Med. Dir. Assoc. 23, 328–329. 10.1016/j.jamda.2021.07.016 35219504

[B17] LeCompte M. D. Schensul J. J. Weeks M. R. Singer M. (1999). “Researcher roles and research partnerships,” in Ethnographer's *Toolkit*, Vol. 6, eds J. J. Schensul and M. D. LeCompte (Walnut Creek, CA: Altamira Press).

[B18] Malinowski B. (2013). Argonauts of the Western Pacific: An Account of Native Enterprise and Adventure in the Archipelagoes of Melanesian New Guinea [1922/1994]. London: Routledge.

[B19] Malta-Müller C. H. Kirkevold M. Martinsen B. (2020). The balancing act of dementia care: an ethnographic study of everyday life and relatives' experiences in a Danish nursing home for people living with advanced dementia. Int. J. Qual. Stud. Health Well-being 15, 1815484. 10.1080/17482631.2020.1815484 32924864 PMC7534205

[B20] Russell L. (2005). It's a question of trust: balancing the relationship between students and teachers in ethnographic fieldwork. Qual. Res. 5, 181–199. 10.1177/1468794105050834

[B21] Sangaramoorthy T. Kroeger K. A. (2020). Rapid Ethnographic Assessments: A Practical Approach and Toolkit for Collaborative Community Research. London: Routledge. 10.4324/9780429286650

[B22] Smit J. P. Hessels L. K. (2021). The production of scientific and societal value in research evaluation: a review of societal impact assessment methods. Res. Eval. 30, 323–335. 10.1093/reseval/rvab00235422392

[B23] Snow D. A. Benford R. D. Anderson L. (1986). Fieldwork roles and informational yield: a comparison of alternative settings and roles. Urban Life. 14, 377–408. 10.1177/0098303986014004002

[B24] Van Maanen J. (2011). Tales of the Field: On Writing Ethnography. Chicago, IL: University of Chicago Press. 10.7208/chicago/9780226849638.001.0001

[B25] Verbeek H. Zwakhalen S. Schols J. Kempen G. Hamers J. (2020). The living lab in ageing and long-term care: a sustainable model for translational research improving quality of life, quality of care and quality of work. J. Nutr. Health Aging. 24, 43–47. 10.1007/s12603-019-1288-5 31886807 PMC6934630

[B26] Vindrola-Padros C. Vindrola-Padros B. (2018). Quick and dirty? A systematic review of the use of rapid ethnographies in healthcare organisation and delivery. BMJ Qual. Saf. 27, 321–330. 10.1136/bmjqs-2017-007226 29263139

[B27] Walker J. C. Hunt C. (2020). Louts and Legends: Male Youth Culture in an Inner-City School. London: Routledge. 10.4324/9781003116332

